# Profiling Non-motor Symptoms in Monogenic Parkinson’s Disease

**DOI:** 10.3389/fnagi.2020.591183

**Published:** 2020-10-30

**Authors:** Xinyao Liu, Weidong Le

**Affiliations:** ^1^Liaoning Provincial Center for Clinical Research on Neurological Diseases, The First Affiliated Hospital, Dalian Medical University, Dalian, China; ^2^Liaoning Provincial Key Laboratory for Research on the Pathogenic Mechanisms of Neurological Diseases, The First Affiliated Hospital, Dalian Medical University, Dalian, China; ^3^Institute of Neurology, Sichuan Academy of Medical Sciences-Sichuan Provincial Hospital, Chengdu, China

**Keywords:** Parkinson’s disease, monogenic, diagnosis, non-motor symptoms, *SNCA*

## Abstract

Parkinson’s disease (PD) is the second most common neurodegenerative disease in the elder population, pathologically characterized by the progressive loss of dopaminergic neurons in the substantia nigra. While the precise mechanisms underlying the pathogenesis of PD remain unknown, various genetic factors have been proved to be associated with PD. To date, at least 23 loci and 19 disease-causing genes for PD have been identified. Although monogenic (often familial) cases account for less than 5% of all PD patients, exploring the phenotypes of monogenic PD can help us understand the disease pathogenesis and progression. Primary motor symptoms are important for PD diagnosis but only detectable at a relatively late stage. Despite typical motor symptoms, various non-motor symptoms (NMS) including sensory complaints, mental disorders, autonomic dysfunction, and sleep disturbances also have negative impacts on the quality of life in PD patients and pose major challenges for disease management. NMS is common in all stages of the PD course. NMS can occur long before the onset of PD motor symptoms or can present in the middle or late stage of the disease accompanied by motor symptoms. Therefore, the profiling and characterization of NMS in monogenic PD may help the diagnosis and differential diagnosis of PD, which thereby can execute early intervention to delay the disease progression. In this review, we summarize the characteristics, clinical phenotypes, especially the NMS of monogenic PD patients carrying mutations of *SNCA, LRRK2, VPS35, Parkin, PINK1, DJ-1*, and *GBA*. The clinical implications of this linkage between NMS and PD-related genes are also discussed.

## Introduction

Parkinson’s disease (PD) is the second most common neurodegenerative disease in the elderly population worldwide. In the 15 most populous countries in the world, more than 4 million people over the age of 50 have PD, and this number is expected to double by 2030 (Dorsey et al., 2007). PD reduces patients’ quality of daily life and causes a heavy economic burden on patients, their families, and the whole society (Meissner et al., 2011). Various pathological mechanisms, such as aging, neuroinflammation, abnormal protein aggregation, mitochondrial dysfunction, oxidative stress, and environmental neurotoxins, have been reported to be associated with the progressive loss of dopaminergic neurons in the substantia nigra of PD brain. However, to date, the exact molecular mechanisms underlying the loss of these neurons remain elusive (Hirsch et al., 2013).

The clinical manifestations of PD involve motor and non-motor symptoms (NMS). Motor symptoms include bradykinesia, rigidity, resting tremor, postural and gait instability, etc., while NMS consists of cognitive decline, autonomic dysfunction, depression, anxiety, sleep disorder, and olfactory impairment (Kalia and Lang, 2015). Since the identification of the first PD-related gene *SNCA* in 1997 (Singleton et al., 2003), at least 23 loci and 19 disease-causing genes for PD have been identified (Deng et al., 2018). According to the recommendations of the Movement Disorder Society Nomenclature of Genetic Movement Disorders, the confirmed forms of monogenic PD with a *PARK* designation can be categorized into those presenting with: (1) classical PD; (2) early-onset PD but clinically similar to nongenetic PD; and (3) atypical parkinsonism (Marras et al., 2016b). In general, autosomal dominant forms are more frequently associated with a phenotype overlapping idiopathic PD (iPD), and autosomal recessive forms with young-onset parkinsonism similar to iPD or parkinsonism with atypical features (Marras et al., 2016b).

NMS usually occurs years to decades before motor symptoms, indicating the PD pathological process may initiate long before the presence of clinical motor symptoms (Pfeiffer, 2016). At present, most studies in PD are still focusing on the improvement of diagnosis and treatment of motor symptoms. The identification of NMS in PD is of significance for the early management of PD. Prompt identification and treatment of these NMS may improve patients’ quality of life (Huang et al., 2019; Seppi et al., 2019). It is worth noting that relatively few studies have specifically targeted the NMS in monogenic PD. Therefore, this review will summarize the characteristics, clinical phenotypes, especially the NMS of monogenic PD patients bearing mutations in various PD-related genes including *SNCA*, the leucine-rich repeat kinase 2 (*LRRK2*), vacuolar protein sorting 35 (*VPS35*), *Parkin*, PTEN-induced putative kinase 1 (*PINK1*), DJ-1 and glucocerebrosidase (*GBA*). The clinical implications of the linkage between NMS and PD-related genes are also discussed.

## PARK1/PARK4-SNCA

In the discovery of monogenic forms of PD, *SNCA* is the first identified and is likely the most intensively investigated gene, although *SNCA* mutations are extremely rare in PD patients (up to 1% in different populations) (Singleton et al., 2003). Misfolded α-synuclein is a major constituent of Lewy bodies and Lewy neurites, which are pathological hallmarks of PD. The contribution of α-synuclein to PD is well established, but the exact molecular mechanisms remain obscure (Hirsch et al., 2013).

Compared with iPD, PD patients carrying *SNCA* mutations are characterized as early-onset, rapid progression, good levodopa response, and obvious NMS (Kasten and Klein, 2013). According to the statistical analysis of 146 *SNCA* mutation carriers, the proportion of male patients is 54%. Most patients develop symptoms after the age of 40 (Trinh et al., 2018). Gene multiplication seems to be more common in the European and Asian populations (Kasten and Klein, 2013). Patients carrying *SNCA* mutations often experience bradykinesia and rigidity, while only about 30% of mutation carriers report resting tremor and postural instability (Kasten et al., 2017).

As for the NMS features of *SNCA* monogenic PD, cognitive decline is the most common symptom, followed by depression, autonomic dysfunction, and other psychotic manifestations (Kasten et al., 2017; Chen et al., 2020). The longitudinal clinical assessments at a 2-year follow-up study conducted by Papadimitriou et al. (2016) have shown that the prominent NMS includes olfactory, autonomic, and cognitive dysfunctions in *A53T* symptomatic and asymptomatic carriers. *SNCA* multiplication might have a gene dosage effect concerning dementia. Fuchs et al. (2007) reported a Swedish family with parkinsonism due to duplication of the *SNCA* gene. Farrer et al. (2004) found early-onset PD and dementia due to triplication of the *SNCA* gene in a Swedish-American family. The proband began to develop the disease at the age of 31, and the disease progressed rapidly with tremor, stiffness, and bradykinesia; at the age of 45, visual hallucinations and auditory hallucinations and delusions appeared; later, the patient developed an intellectual disability and severe dementia, then died at the age of 52 (Farrer et al., 2004). Dan et al. (2016) showed that *SNCA Rep-1* is related to depression in PD through the study of the genotype and depression performance in 1,134 Chinese PD patients. Recently, Rotter et al. (2019) reported that *SNCA* mRNA expression was positively correlated with the severity of depressive symptoms. The evaluation of dysautonomia including cardiovascular dysfunction was reported in six *SNCA* mutations and was confirmed on neuroimaging and/or neuropathology (Singleton et al., 2004). Both missense mutations, as well as α-synuclein multiplications, have been associated with various degrees of dysautonomia, including in asymptomatic carriers, preceding the onset of motor symptoms (Singleton et al., 2004; Kara et al., 2014). In familial PD with multiplied *SNCA*, although the triplications lead to an earlier and more severe phenotype, *SNCA* duplication may sometimes represent variable degrees of autonomic dysfunction (such as orthostatic hypotension, urinary incontinence, severe constipation, etc) (Kruger et al., 2001). Another study showed that hyposmia is associated with rapid eye movement sleep behavior disorder (RBD) in PD patients carrying *SNCA* mutations (Li et al., 2017). A recent study by Carmona-Abellan et al. (2019) found that *E46K-SNCA* carriers had moderate to severe p-α-synuclein deposits and small-fiber neurodegeneration including nerve fascicles and glands in epidermal and dermal layers. The severity of the latter skin abnormalities in *E46K-SNCA* was correlated with sudomotor dysfunction in hands (Carmona-Abellan et al., 2019). Tambasco et al. (2016) summarized the NMS in *SNCA* point mutations (PMc), duplication (Dc), and triplication (Tc) carriers. They found that among the reported 137 cases, depression is more common in Tc (69.0%) and Dc (35.0%) than in PMc (13.2%) (Tambasco et al., 2016). The frequency of anxiety (Tc 3.4%, Dc 5.0%, and PMc 5.9%) and sleep disturbances (Tc 20.7%, Dc 17.3%, and PMc 10.3%), usually presenting as RBD, manifest with a similar frequency for all 3 groups (Tambasco et al., 2016). Also, 69% of Tc, 42.5% Dc, and 25% of PMc have cognitive impairment (Tambasco et al., 2016). Furthermore, psychosis is more frequent among patients with Tc (69.0%) than those with either Dc (40%) or PMc (7.3%) (Tambasco et al., 2016). Gastrointestinal tract disturbances and urinary dysfunctions are more frequent in Tc (24.1%) than in Dc (12.5%) and PMc (10.3%) (Tambasco et al., 2016). Postural hypotension is present in 37.9% of Tc, 25% of Dc, and 17.6% of PMc in general; but the *E46K* and *G51D* PMc have high incidences of postural hypotension and falls, 44.4% and 37.5%, respectively. Hyposmia is less common (6.9%-22.2%) in PD patients carrying *SNCA* mutations (Tambasco et al., 2016).

## PARK8-LRRK2

In autosomal dominant PD, mutations in the *LRRK2* gene are the most common cause of monogenic PD (Paisán-Ruiz et al., 2004). Autosomal dominant missense mutations in the *LRRK2* gene account for 1% to 2% of all PD cases, a higher proportion among Ashkenazi Jews and North African Berbers (Paisán-Ruiz et al., 2013). Currently, the detailed function and pathogenicity of *LRRK2* in PD are not fully understood. Rab GTPase family members are cellular physiological substrates of *LRRK2* kinase (Jeong et al., 2018). Studies of the relationship between *LRRK2* and Rab protein members Rab7, Rab5B, and Rab29 have demonstrated that *LRRK2* pathogenic mutations dysregulate vesicle trafficking (Shin et al., 2008; Dodson et al., 2012; MacLeod et al., 2013). Many Rab proteins including Rab3A/B/C/D, Rab8A/B, Rab10, Rab12, Rab35, Rab43, Rab5B/C, and Rab29 are the substrates of *LRRK2* kinase (Steger et al., 2017). *LRRK2*-mediated Rab35 phosphorylation positively regulates α-synuclein propagation (Bae et al., 2018). *LRRK2* also affects mitochondrial morphology and function and regulates autophagy, both etiologic factors in PD (Rosenbusch and Kortholt, 2016; West and Cookson, 2016). Moreover, recent studies have further demonstrated a higher level of *LRRK2* in immune cells, including circulating B lymphocytes, dendritic cells, and macrophages, suggesting the involvement of *LRRK2* in the immune system (Hakimi et al., 2011). More than 40 mutations in *LRRK2* have been found, of which eight (*N1437H, R1441C/G/H/S, Y1699C, G2019S*, and *I2020T*) cause PD and *A419V, R1628P, M1646T* and *G2385R* are considered risk factors for PD (Heckman et al., 2013; Li et al., 2015; Mata et al., 2017; Alessi and Sammler, 2018; De Wit et al., 2018).

Of all the *LRRK2* mutations, the best-described one is *G2019S*, with a penetration rate from 28% at the age of 59 to 74% at the age of 79, which is indistinguishable from that of iPD (Aasly et al., 2005; Healy et al., 2008). *LRRK2* mutations typically manifest as late-onset PD, and the *LRRK2* carriers have a good response to levodopa (Deng et al., 2005). PD patients with *LRRK2* mutations often show postural instability gait difficulty phenotype compared with noncarriers, and progress at a rate similar to iPD in a longitudinal study (Nabli et al., 2015). The prospective longitudinal follow-up study of PD patients with or without the *LRRK*2 *G2019S* mutation by Saunders-Pullman et al. (2018) from the cross-sectional study, shows a slower decline in motor UPDRS scores among those PD patients with *LRRK*2 *G2019S*.

*LRRK2* monogenic PD patients have fewer NMS than iPD. vs. iPD patients, monogenic PD patients carrying *LRRK2-G2019S* mutation have relatively better performance in attention, executive function, and language domains (Alcalay et al., 2015b). In other studies, it is reported that patients with *LRRK2* monogenic PD also perform better than iPD in terms of cognitive ability, smell, sleep, and emotion (Marras et al., 2011, 2016a; Ben Sassi et al., 2012; Saunders-Pullman et al., 2014, 2015). But dementia also has been reported in PD patients carrying *LRRK2* mutation (Tomiyama et al., 2006). Lohmann et al. (2009) reported a comparable rate of depression between PD patients carrying *G2019S* mutation and unaffected *G2019S*-carriers, and the prevalence of anxiety is similar between patients with iPD and those *G2019S*-carriers. However, mice expressing human *LRRK*2 *G2019S* exhibit mild cognitive impairment earlier than motor dysfunctions (Adeosun et al., 2017). Higher UPDRS motor scores, more frequent urinary problems, and fewer hours of sleep are found in mutation carriers compared to non-carriers (Johansen et al., 2011). The mutation carriers with UPDRS ≥8 are all aged over 50 years and have shorter overall sleeping hours, more frequent urinary and constipation problems, as well as higher mood scores and body mass index (Johansen et al., 2011). Furthermore, RBD, based on questionnaires or polysomnography, appears to be uncommon in *LRRK2* mutation carriers (Ehrminger et al., 2015; Saunders-Pullman et al., 2015).

## PARK17-VPS35

The PD-related mutations of the *VPS35* gene were firstly identified in 2011 (Vilarino-Guell et al., 2011). In 2012, a large multi-center study involving 15,000 individuals worldwide estimated that *PARK-VPS35* mutations account for approximately 0.4% of PD (Sharma et al., 2012). *PARK-VPS35* monogenic PD has high heritability and low penetrance, and its phenotype is very similar to that of iPD, but the average age of onset is 50 years old (Sharma et al., 2012). In an autosomal dominant PD population study in Japan, *VPS35* mutation is also very rare, and the phenotype of motor symptoms in this study is prominent tremor (Ando et al., 2012). *VPS35* is a key molecule in the retromer complex within the cell in terms of regulating endosomal trafficking (Cutillo et al., 2020). However new data suggest that *VPS35* also regulates mitochondrial dynamics and homeostasis (Cutillo et al., 2020). Also, *VPS35* is a critical player in pathways connected to α-synuclein accumulation and clearance (Cutillo et al., 2020).

The NMS profile in *VPS35* monogenic PD is similar to that in iPD, including hyposmia, rare mild cognitive impairment, and rare neuropsychiatric features (Struhal et al., 2014). The autonomic manifestations of *VPS35* monogenic PD, such as orthostatic hypotension and constipation, have also been reported (Yamaguchi et al., 2005).

## PARK2-Parkin

*Parkin* mutations are the most common cause of autosomal recessive PD, and the median age at onset is 31 years (Bruggemann and Klein, 1993). *Parkin* is an E3 ubiquitin ligase protein that catalyzes the transfer of ubiquitin to its specific target protein (Narendra et al., 2012). In one meta-analysis of early-onset PD, Kilarski et al. (2012) found a familial mutation frequency of 15.5%, while the mutation rate in the sporadic was 4.3%. At least 60 *Parkin* mutations and variants have been identified, which may vary with specific mutations and presents a major challenge in determining pathogenicity (Hedrich et al., 2004). Also, deletions and duplications of *Parkin* are particularly common, which complicates *Parkin* genotyping (Klein et al., 2000).

The clinical characteristics of *Parkin* monogenic PD are early-onset and slow progression with a good response to dopaminergic treatment, but are usually complicated by dystonia and prominent freezing of gait, with sleep benefit on most symptoms (Ishikawa and Tsuji, 1996; Grunewald et al., 2013).

Overall, NMS appears to be less severe in patients with *Parkin* monogenic PD than in iPD. Compared with iPD or PD patients with *Parkin* heterozygotes, PD patients with *Parkin* homozygotes and compound heterozygotes may have less olfactory dysfunction (Khan et al., 2004). A follow-up study (Consortium on Risk for Early-Onset PD) examined the cognitive function in early-onset PD with the long-duration disease (>14 years) and found that *Parkin* carriers performed better on the Mini-Mental State Examination, clinical dementia ratings, attention, memory, and visuospatial performance than noncarriers (Alcalay et al., 2014). Psychosis has been rarely reported in *Parkin* carriers, and if occurs it is usually before motor symptom onset. Psychotic symptoms are usually related to dopamine replacement therapy (Khan et al., 2003; Kim et al., 2014). However, *Parkin* carriers may have more serious impulse control disorders, frequent compulsive shopping, overeating, and punding/hobbyism (Morgante et al., 2016). The score of the Questionnaire for Impulsive-Compulsive Disorders in PD–Rating Scale in patients with *parkin*-PD is higher than nonmutated PD patients (Morgante et al., 2016). In some other studies, the frequency of RBD in *Parkin*-related PD is no different from iPD. However, *Parkin* carriers are more likely to have restless leg syndrome than iPD (Kumru et al., 2004; Limousin et al., 2009). Tijero et al. (2015) found that the rate of autonomic symptoms in *Parkin*-related PD is much lower than iPD, and the heart noradrenergic innervation is relatively preserved.

## PARK6-PINK1

Mutations in the *PINK1* gene are the second most common cause of autosomal recessive early-onset PD after *Parkin* (Valente et al., 2004a). According to previous reports, the frequency of *PINK1* mutations in sporadic early-onset PD is 4% to 7% (Valente et al., 2004b; Bonifati et al., 2005). *PINK1* is a serine/threonine kinase containing key regulatory sites, which activates ubiquitin by phosphorylation and cooperates with the downstream ubiquitin ligase *PARKIN*, to exert quality control and regulate autophagic degradation of mitochondria and misfolded proteins in all cell types (Valente et al., 2001; Truban et al., 2017). Recent research shows *PINK1* deficiency as an early modulator of innate immunity in neurons, which precedes the late stages of neuroinflammation during α-synuclein spreading (Torres-Odio et al., 2017).

*PINK1* monogenic PD progresses slowly with a 32-year average age of onset, good levodopa response, and sleep benefit. The most common motor symptom of *PINK1* monogenic PD is bradykinesia followed by rigidity (Valente et al., 2004b; Bonifati et al., 2005). The *PINK1* monogenic PD patients occasionally have pyramidal signs or hyperreflexia (Kasten et al., 2018).

It has been reported that psychiatric symptoms such as anxiety and depression are more prominent in *PINK1*-linked PD (Ephraty et al., 2007). According to a recent study, the incidence of cognitive dysfunction is higher in *PINK1* mutations (29.4%), and executive function and attention are most affected (Piredda et al., 2020). Regarding the mental symptoms, depression is the most common one (37.5%; Piredda et al., 2020). In a research survey of 20 members of a family (four homozygous, 11 heterozygous, and five non-mutation carriers), Steinlechner et al. (2007) found that 61% mutation carriers and 20% non-mutation carriers have predominantly affective and schizophrenia spectrum disorders. Affective and psychotic symptoms may be part of the phenotypic spectrum or even the sole manifestation of patients with *PINK1* mutations (Funayama et al., 2008). Hyposmia appears to be a common NMS in patients with *PINK1* monogenic PD (Ferraris et al., 2009). A review of the clinical phenotype of *PINK1*-related PD cases confirm that autonomic symptoms are not common; among them, the urinary dysfunction consists of 44%, and orthostatic hypotension consists of 22% (Chaudhuri et al., 2006). Ricciardi et al. (2014) conducted a 12-year follow-up study in 5 *PINK1* homozygous and 14 heterozygous mutation carriers from two large Italian families. They found all but one patient has sleep impairment, impulse control disorders, anxiety, apathy, and global cognitive impairment (Ricciardi et al., 2014).

## PARK7-DJ-1

The mutation in *DJ-1* was firstly identified in two close relative families from the Netherlands and Italy (Bonifati et al., 2003), with a very low frequency of mutation. *DJ-1* mutation accounts for less 1% of early-onset PD (Sironi et al., 2013). The meta-analysis by Kilarski et al. (2012) found that the overall mutation frequency of *DJ-1* was 0.4%, which was slightly higher in familial PD (0.8%) than sporadic PD (0.4%). The median age of disease onset is 27 years (Weissbach et al., 2019).

The clinical manifestations of patients with *DJ-1* mutations are similar to those with *Parkin* or *PINK1* mutations. However, the NMS in PD patients carrying *DJ-1* mutation is more prominent, including mental disorders and cognitive decline (Kilarski et al., 2012; Kasten et al., 2018). Dystonia is particularly common (16/22; 73%) in *DJ-1* mutation carriers, as well as the postural tremor (8/12; 67%), and psychotic signs (8/11; 73%; Weissbach et al., 2019). All but one of the described patients suffer from depression, suggesting that psychotic features may be particularly common among *DJ-1* mutations carriers and may be more common than in iPD (Weissbach et al., 2019).

## GBA

It is often presented as Gaucher’s disease (GD) with PD in an aggressive phenotype, in which both homozygous and heterozygous *GBA* mutations seem to be prone to PD (Zhao et al., 2016). *GBA* mutations become the most important genetic susceptibility factor for PD (Zhao et al., 2016). Multiple studies have established a significantly increased risk of PD associated with *GBA* mutations (Sidransky et al., 2009; Huang et al., 2011). Studies have shown that PD cases with *GBA* mutations are on average 5 years younger than iPD (Clark et al., 2007; Sidransky et al., 2009). Penetrance is incomplete and increases with age (Gan-Or et al., 2015a). *GBA* encodes the lysosomal enzyme glucocerebrosidase (GCase), which cleaves the β-glucosyl linkage of glucosylceramide and glucosylsphingosine (Beavan and Schapira, 2013). Alcalay et al. (2015a) found that lower GCase activity may be associated with a faster course of disease progression.

Since glucosylceramide, the substrate of GCase, is mainly stored in cells of the reticuloendothelial system, macrophages carrying matrix-filled lysosomes (the classic cell marker of GD) accumulate in the spleen and liver when *GBA* is mutated. Organ enlargement and inflammation occur, and sometimes the nervous system is also involved. According to the clinical progress of GD and the speed of nervous system involvement, GD is described as three types (Grabowski, 2008). Type I GD is classically defined as a non-neurological pathology (the N370 mutation is the most common) with its typical features of hepatosplenomegaly, bone and hematopoietic system abnormalities (Bultron et al., 2010). Type II and type III GD (type *L444P* mutation is the most common, point mutations or complex allelic mutations can occur) can be distinguished from type I GD by whether there is any neurodegeneration in the central nervous system. While the development of Type II GD is fast, the development of Type III GD is slow (Mazzulli et al., 2011).

The characteristic clinical phenotype of *GBA* monogenic PD is similar to those of sporadic PD, but the onset is earlier and the course is more severe (Brockmann et al., 2015; Ryan et al., 2019). Furthermore, PD patients with *GBA* mutations usually have an earlier and more frequent occurrence of NMS (Petrucci et al., 2020). It is worth noting that the NMS in PD patients carrying *GBA* mutations seems to be more prominent than iPD (Sidransky et al., 2009). Studies have shown that *GBA* mutations can cause more serious cognitive impairments, characterized by greater declines in working memory, executive function, and visual-spatial ability (Mata et al., 2016). Previous studies suggested that 45% of *GBA* monogenic patients have visual hallucinations unrelated to dopamine therapy (Neumann et al., 2009). However, a high frequency (78%) of visual hallucinations under dopamine replacement therapy has also been reported in *GBA*, higher than that of *LRRK2* (38%) or iPD (53%) (Wang et al., 2014). Similar results have been found in other studies, with rates of psychosis in *GBA* patients receiving dopamine replacement therapy ranging between 46% and 53% (Oeda et al., 2015; Yahalom et al., 2019). Finally, a recent meta-analysis revealed that *GBA* is associated with a 1.83-fold increase in the risk of mental illness (Creese et al., 2018). After onset, *GBA* has visual hallucinations, often accompanied by cognitive decline or dementia (Neumann et al., 2009; Oeda et al., 2015). The prevalence of depression (33%) in PD patients with *GBA* mutation, especially in male patients, is also significantly higher than that in iPD (13%) (Swan et al., 2016). The impact of *GBA* mutation on olfactory in PD is similar to that in iPD (Alcalay et al., 2012), whereas autonomic dysfunction may be more common (Brockmann et al., 2011). Also, *GBA* mutations are associated with RBD in PD patients (Gan-Or et al., 2015b). Thaler et al. (2017) recently reported a gene-dose effect, in which homozygotes and compound heterozygotes have earlier disease onset and more severe motor and NMS compared with both heterozygotes and iPD.

## Conclusion

The exploration of PD has never stopped in recent years. Although gene mutations only exist in a small portion of PD patients, genetic studies have greatly promoted the biggest advances in PD research. In the observational study of PD, the clinical phenotype of different gene mutations has different characteristics. NMS is very common and usually appear earlier than motor symptoms of PD. Different gene mutations are also characterized by various NMS. Such as PD patients with SNCA mutations have a relatively common cognitive decline. *LRRK2* mutations in late-onset PD present less NMS than iPD with a similar rate of progression. NMS in patients with *PARKIN* mutations are not as severe as iPD. The mental symptoms of *PINK1* monogenic PD are more frequent, including anxiety and depression. NMS in *DJ-1* monogenic PD is more prominent, including depression, anxiety, and other mental illness and cognitive decline. The PD patients carrying *GBA* mutations usually present with a high frequency of cognitive disorders, such as impaired working memory, executive function, and visual-spatial ability. Also, the incidence of anxiety and depression is high, and autonomic dysfunction may be more severe in GBA monogenic PD (Table 1). The profiling and characteristics of NMS in monogenic PD should be helpful for the early diagnosis and clinical management of the disease.

**Table 1:** Phenotypes of different monogenic Parkinson’s disease with motor and non-motor symptoms.

Gene	Disease onset	Inheritance	Motor symptoms	Non-motor symptoms
*PARK1/PARK4-SNCA*	Early	Autosomal dominant	Rapid progression, good levodopa response; most often experience bradykinesia and rigidity; only about 30% report resting tremor and postural instability.	Cognitive decline is the most common, followed by depression, autonomic symptoms, and psychotic symptoms; includes olfactory disorders.
*PARK8-LRRK2*	Late	Autosomal dominant/autosomal recessive	Good response to levodopa; postural instability; gait difficulty phenotype; progressed at a rate similar to iPD.	Fewer nonmotor manifestations than iPD.
*PARK17-VPS35*	Early	Autosomal dominant	Phenotype similar to iPD; Case reports only Tremor-predominant PD.	Non-motor symptoms similar to iPD.
*PARK2-Parkin*	Juvenile or early	Autosomal recessive	Slow progress; good response to dopaminergic treatment, usually complicated by dystonia and prominent freezing of gait; with sleep benefit on most symptoms.	Less severe than in iPD; less olfactory dysfunction; performed better on the Mini-Mental State Examination, clinical dementia rating, attention, memory, and visuospatial performance; but may have more serious impulse control disorders.
*PARK6-PINK1*	Juvenile or early	Autosomal recessive	Progresses slowly; levodopa responds well and persistent; sleep benefit; the common symptom is bradykinesia and rigidity; fewer pyramidal signs or hyperreflexia.	Psychiatric symptoms are more prominent; include anxiety and depression, but cognitive impairment is less involved; Hyposmia seems to be common.
*PARK7-DJ-1*	Juvenile or early	Autosomal recessive	Good levodopa response; dystonia is particularly common; others include postural tremor.	The non-motor symptoms are more prominent, including depression, anxiety, and other mental illness, cognitive decline.
*GBA*	Early	Autosomal dominant	Similar to iPD, but the onset is earlier and the course is more serious.	Non-motor symptoms are more prominent than iPD; cause more serious cognitive impairments, working memory, executive function, and visual-spatial ability. The incidence of anxiety and depression is high, autonomic dysfunction may be more severe, and olfactory disturbance is similar to iPD; are associated with idiopathic RBD and possible RBD in PD patients.

## Author Contributions

XL and WL contributed to the review of the available literature. XL drafted the manuscript. WL revised the text to the final form. All authors contributed to the article and approved the submitted version.

## Conflict of Interest

The authors declare that the research was conducted in the absence of any commercial or financial relationships that could be construed as a potential conflict of interest.
